# Hypoxia Enhances Differentiation of Adipose Tissue-Derived Stem Cells toward the Smooth Muscle Phenotype

**DOI:** 10.3390/ijms19020517

**Published:** 2018-02-08

**Authors:** Fang Wang, Vladimir Zachar, Cristian Pablo Pennisi, Trine Fink, Yasuko Maeda, Jeppe Emmersen

**Affiliations:** 1 Laboratory for Stem Cell Research, Aalborg University, Fredrik Bajers Vej 3B, 9220 Aalborg, Denmark; wangfang1995@hotmail.com (F.W.); vlaz@hst.aau.dk (V.Z.); cpennisi@hst.aau.dk (C.P.P.); trinef@hst.aau.dk (T.F.); 2 Sir Alan Parks Physiology Unit, St. Mark’s Hospital, Northwick Park, Watford Road, Harrow HA1 3UJ, UK; yazmaeda@gmail.com

**Keywords:** mesenchymal stem cell, human adipose tissue-derived stem cell, smooth muscle cell, differentiation, hypoxia, oxygen

## Abstract

Smooth muscle differentiated adipose tissue-derived stem cells are a valuable resource for regeneration of gastrointestinal tissues, such as the gut and sphincters. Hypoxia has been shown to promote adipose tissue-derived stem cells proliferation and maintenance of pluripotency, but the influence of hypoxia on their smooth myogenic differentiation remains unexplored. This study investigated the phenotype and contractility of adipose-derived stem cells differentiated toward the smooth myogenic lineage under hypoxic conditions. Oxygen concentrations of 2%, 5%, 10%, and 20% were used during differentiation of adipose tissue-derived stem cells. Real time reverse transcription polymerase chain reaction and immunofluorescence staining were used to detect the expression of smooth muscle cells-specific markers, including early marker smooth muscle alpha actin, middle markers calponin, caldesmon, and late marker smooth muscle myosin heavy chain. The specific contractile properties of cells were verified with both a single cell contraction assay and a gel contraction assay. Five percent oxygen concentration significantly increased the expression levels of α-smooth muscle actin, calponin, and myosin heavy chain in adipose-derived stem cell cultures after 2 weeks of induction (*p <* 0.01). Cells differentiated in 5% oxygen conditions showed greater contraction effect (*p <* 0.01). Hypoxia influences differentiation of smooth muscle cells from adipose stem cells and 5% oxygen was the optimal condition to generate smooth muscle cells that contract from adipose stem cells.

## 1. Introduction

Smooth muscle cell (SMC) is one of the major components of the human body and plays an important role in the normal function maintenance of respiratory, gastrointestinal, and genitourinary systems. In a gastrointestinal tract, circular and longitudinal smooth muscles in the wall are essential to transport, digest, and absorb food as well as expel the waste [1]. The internal anal sphincter is a continuation of intestinal smooth muscle encircling the anal canal, which is thin (2–3 mm) but a crucial barrier to involuntary loss of stool. The function of smooth muscle can be weakened due to congenital abnormality, or impairment could develop due to trauma such as obstetric injuries, surgical operations, or degeneration with aging.

The use of regenerative medicine and tissue engineering is an emerging and promising alternative for tissue repair. Regeneration of smooth muscle with autologous stem cell could be ideal to avoid any immunogenic reaction. In addition, it has the potential of restoring functional contraction and structural integrity of the intestinal smooth muscle, which cannot be achieved with surgical repair, which is the case for the internal anal sphincter [2,3]. 

Several groups have reported that smooth muscle cells (SMCs) could be differentiated either from embryonic stem cells or bone marrow-derived mesenchymal stem cells. However, both types of stem cells have their limitations, as the former has inherent ethical issues and the latter has expansion capacity limitations, which make them both difficult to meet increasing stem cell-based treatment. In contrast, adipose tissue-derived stem cells (ASCs) are easily accessible and abundant in number and are considered an attractive stem cell source for regenerative medicine and tissue engineering [4]. Several studies have reported successful differentiation of ASCs into smooth muscle cells, using a variety of differentiation factor such as transforming growth factor beta 1 (TGF-β1) and bone morphogenetic protein 4 (BMP4) alone and in combination, as well as heparin and angiotensin II, probably by activating the TGF-β receptor dependent Smad2 pathway [5,6,7].

Thus, the family of transforming growth factor (TGF)-related proteins emerges as the most potent set of soluble growth factors promoting SMCs differentiation. TGF-β1 is a multifunction protein that plays critical roles in a variety of biological processes including cell growth, differentiation, and migration [8]. Bone morphogenetic proteins represent the largest group in the TGF cytokine superfamily. Lagna et al. reported that the bone morphogenetic protein (BMP) signaling pathway effectively induced contractile phenotype and SMC-specific genes transcription [8]. Nuclear localization and recruitment of the myocardin-related transcription factor-A (MRTF-A) and myocardin-related transcription factor-B (MRTF-B) to a smooth muscle α-actin promoter was also observed in response to BMP [8]. In addition, the combination of TGF-β1 (5 ng/mL) and BMP4 (2.5 ng/mL) stimulation for 1 week drove the ASCs into mature contractile SMCs [3]. Similarly, TGF-β1 and BMP4 were shown to reduce SMC proliferation and migration and promote the expression of SMC contractile genes [4]. The study from Lagna et al. provided evidence that a SMC phenotypic switch induced by BMP4 from synthetic to contractile was mediated by a Smad and RhoA/Rho kinase-dependent and TGF-β receptor-independent signaling pathway. The BMP pathway activated transcription of SMC genes by inducing nuclear translocation of the transcription factors MRTF-A and MRTF-B, binding the CArG box of specific gene promoters [8].

One of the challenges in the development or generation of SMC from ASCs is to optimize microenvironments for efficient linear-specific differentiation, cell proliferation, and maintenance of contractile phenotype. Oxygen is an important component of the optimal microenvironment [9]. Although most cell cultures and expansions are performed in vitro at 20% oxygen concentration, the actual oxygen tensions of many tissues in physiological environment are much lower: oxygen concentration in adipose tissue is approximately 2–8% [10]. Furthermore, the oxygen concentration sensed by cells ranges from 1% to 5% under normal physiological conditions [11]. The impact of hypoxia on proliferation and maintenance of stemness of ASCs have been shown consistently: 1% oxygen promotes the maintenance of stemness of ASCs, and 2% and 5% oxygen enhance the proliferation ability of ASCs [12,13,14]. Establishing the optimal oxygen condition to produce functional SMCs from ASCs is an important factor in smooth muscle cell production.

The primary aim of this study was to investigate the effect of oxygen concentrations on the differentiation of SMCs from ASCs.

## 2. Results

### 2.1. Changes in Gene Expression of SMC Marker Genes during Differentiation

**Figure 1:**
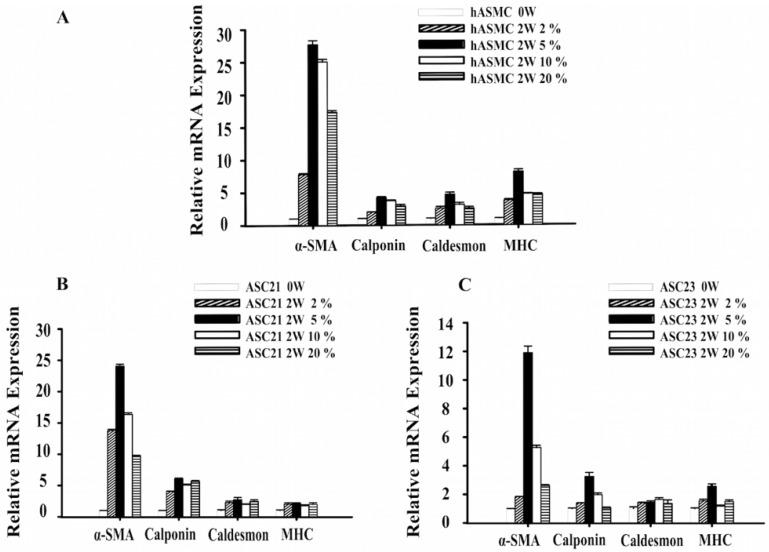
Effect of hypoxia on the expression of smooth muscle cell (SMC)-specific genes before differentiation (0 W) and after 14 days differentiation (2W). (**A**) Expression of SMC-specific genes in SMC. (**B**) Expression of SMC specific genes in ASC21. (**C**) Expression of SMC specific genes in ASC23. Values were expressed as mean ± SE. Analysis of variance showed the overall model to be significant (*p <* 0.01). Both oxygen levels and time of differentiation were significantly different in the post hoc analysis (*p <* 0.01 for both factors). α-SMA: alpha-smooth muscle actin; MHC: myosin heavy chain.

The positive control, human aortic smooth muscle cells (SMCs), showed increased expression of the differentiation markers alpha-smooth muscle actin (α-SMA), calponin, caldesmon, and myosin heavy chain (MHC) after differentiation for 2 weeks in all four oxygen conditions (Figure 1A). The early marker α-SMA was increased to 27-fold when differentiated at 5% oxygen compared to undifferentiated SMCs (*p <* 0.01). Myosin heavy chain, as a specific end-point marker of SMC differentiation, was increased eight-fold at the 5% oxygen level (*p <* 0.01).

The change of transcriptional levels in the ASC21 and ASC23 cell cultures are seen in Figure 1B,C. Similar to the SMC results, 5% oxygen concentration significantly increased the expression levels of α-SMA, calponin, and MHC in both the ASC21 and ASC23 cell cultures after 2 weeks of induction (*p <* 0.01). The expression of the middle marker caldesmon gene showed an increasing trend after differentiation for 2 weeks compared to that of undifferentiated ASCs (*p <* 0.01).

### 2.2. Morphological Changes of SMCs and ASCs

**Figure 2:**
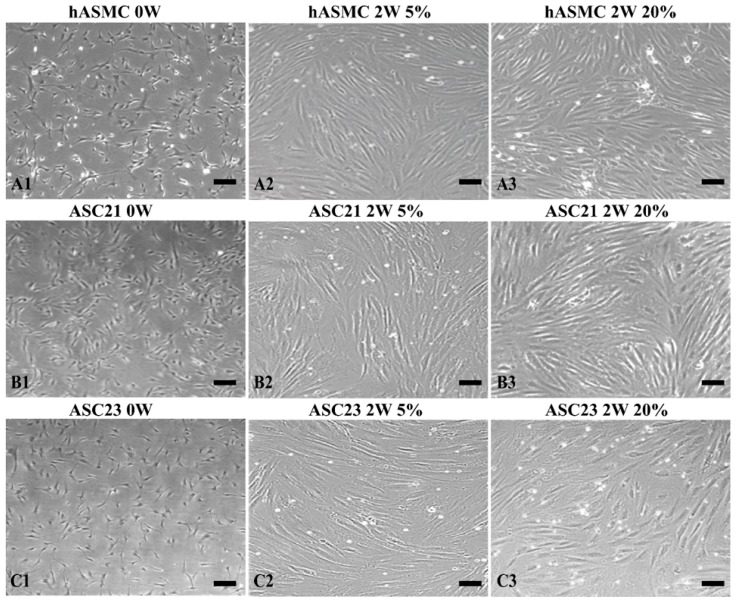
Morphological changes of SMCs and adipose tissue-derived stem cells (ASCs). (**A1**) SMC cultured in proliferation medium for 7 days. (**B1**,**C1**) ASC 21 and 23 cultured in proliferation medium for 7 days. (**A2**,**A3**) SMC induced with smooth muscle differentiation supplement for 2 weeks in 5% or 20% O_2_. (**B2**,**B3**,**C2**,**C3**) ASC 21 and 23 induced with 5 ng/mL transforming growth factor beta 1 (TGF-β1) and 2.5 ng/mL bone morphogenetic protein 4 (BMP4) in combination for 2 weeks in 5% or 20% O_2_. Bar scales: 50 µm for all images.

Human aortic smooth muscle cells, ASC cell cultures 21 and 23, were cultured in proliferation medium to sub-confluence levels for approximately 7 days, and subsequently differentiated for 2 weeks in 2%, 5%, 10%, or 20% oxygen_._ Human aortic smooth muscle cells showed slender stellate shape before differentiation and the two ASC cell cultures exhibited fibroblast-like shape (Figure 2(A1,B1,C1)). After differentiation for 2 weeks, the cellular morphology of SMC switched from small stellate to spindle-like shape (Figure 2(A2,A3)). Similarly, the ASC cell cultures acquired SMC morphology and showed spindle-like morphology and the typical “hill and valley” pattern after differentiation for 2 weeks (Figure 2(B2,B3,C2,C3)). 

### 2.3. Effect of Hypoxia on Differentiation of Cells at the Protein Level

**Figure 3:**
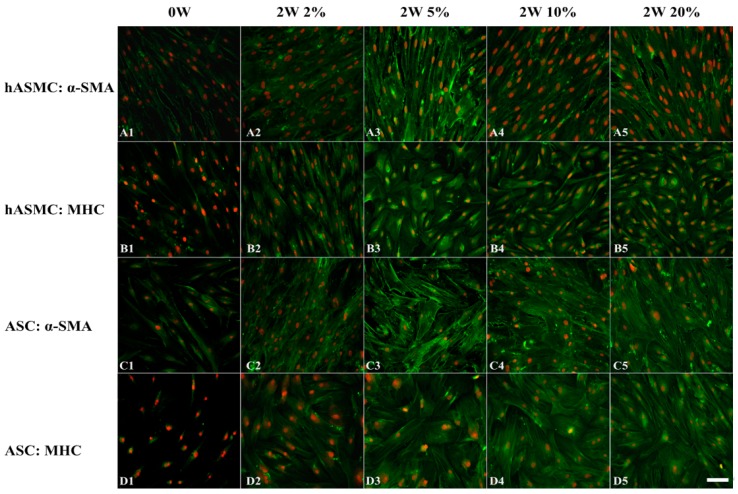
Effect of hypoxia on the expression of SMC-specific proteins. (**A1**–**A5**) Expression of α-SMA in undifferentiated and differentiated SMCs. (**B1**–**B5**) Expression of MHC in undifferentiated and differentiated SMCs. (**C1**–**C5**) Expression of α-SMA in undifferentiated and differentiated ASC23. (**D1**–**D5**) Expression of MHC in undifferentiated and differentiated ASC23. Bar scales: 20 μm for all images. Alpha-SMA and MHC in green; nuclei in red.

To investigate the effect of hypoxia on ASC differentiation, the smooth muscle-specific contractile proteins α-SMA and MHC were visualized by immunofluorescence staining. There were baseline expressions of α-SMA and MHC in undifferentiated SMCs and only α-SMA was observed in undifferentiated ASCs. Differentiated SMCs showed a remarkable increase in two kinds of protein expression levels compared to SMCs at week 0 when placed in oxygen concentrations of 2%, 5%, 10%, and 20% O_2_ (Figure 3(A1–A5,B1–B5)). When both ASC lines were induced by TGF-β1 and BMP4 in combination, α-SMA and MHC were expressed in all oxygen concentrations (Figure 3(C1–C5,D1–D5)). 

### 2.4. Dynamic Contraction Process of Differentiated Stem Cells

To verify the ability of differentiated ASCs to contract by induction with carbachol, a single-cell contraction assay was conducted at 20% O_2_.

**Figure 4:**
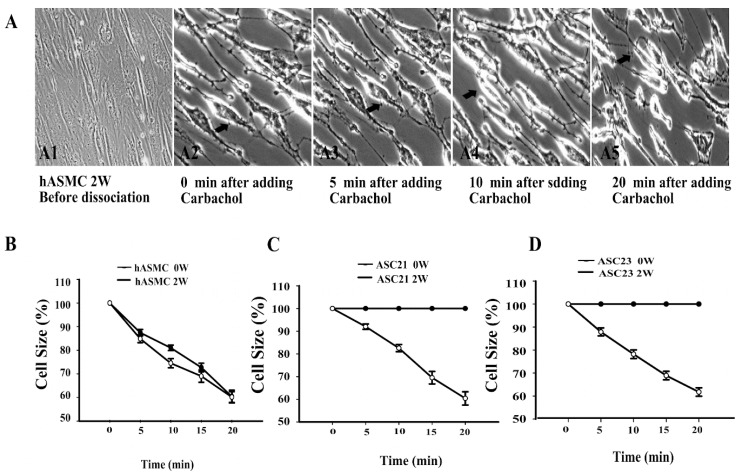
Analysis of contractility of SMCs and ASCs. (**A1**) SMCs 2 weeks before treatment with non-enzymatic dissociation buffer. (**A2**–**A5**) SMCs 2 weeks after treatment with non-enzymatic dissociation buffer and carbachol (100 µM). (**B**) Quantitative analysis of contractive ability of SMCs before (0 W) and after differentiation for 2 weeks (2 W). (**C**) Contraction of ASC21 before (0 W) and after differentiation (2 W). (**D**) Contraction of ASC23 before (0 W) and after differentiation (2 W). Arrows indicate contraction of the same SMC in different time points after the addition of carbachol. Data is expressed as means ± SE. Analysis of variance showed the overall model to be significant (*p <* 0.01, F = 111.99). Post hoc analysis showed both cell cultures and state of differentiation to be significantly different from each other (*p <* 0.01 for both).

As shown in Figure 4, both undifferentiated and differentiated SMCs and ASCs were dissociated into separate, semi-adherent cells when the culture medium was replaced with a non-enzymatic cell dissociation buffer at 37 °C for 30 min. This allowed the cells to contract while remaining in contact with the rigid plastic surface of the culture well. Figure 4(A1,A2) shows the sub-confluence and relaxed state of differentiated SMCs before detachment and contraction. By contrast, Figure 4(A3–A5) shows a decrease of cell sizes at different time points after treatment with carbachol. When treated with 100 µM carbachol, the SMCs started contraction immediately, as shown in Figure 4(A3). The cell sizes were further decreased with the whole contraction process (Figure 4(A3–A5)). In order to compare the contractile ability of different cell cultures, the cell sizes were measured before and after dissociation, and at 5, 10, and 20 min after stimulation with carbachol. When SMCs were stimulated by carbachol, initial responses were faster for week 2 cells compared to week 0 cells (Figure 4B). For ASC21 and ASC23, undifferentiated week 0 cells did not contract (Figure 4C,D). The mean cell size after contraction in all of the differentiated cell cultures was approximately 60% of original size (SMC = 60.2%; ASC21 = 60.4%; ASC23 = 61.7%, *p <* 0.01).

### 2.5. Effect of Hypoxia on Contractile Ability of SMCs Differentiated Form ASCs by Collagen Gel Lattice Assay

To examine the physiological effect of hypoxia on differentiation of ASC, a collagen gel contraction assay was used, which measures the change in gel size over time. Based on the gene expression results, we limited the assay to investigate contraction of cells grown at 5% and 20% O_2_ and used undifferentiated (week 0) cells from each cell culture as controls. 

**Figure 5:**
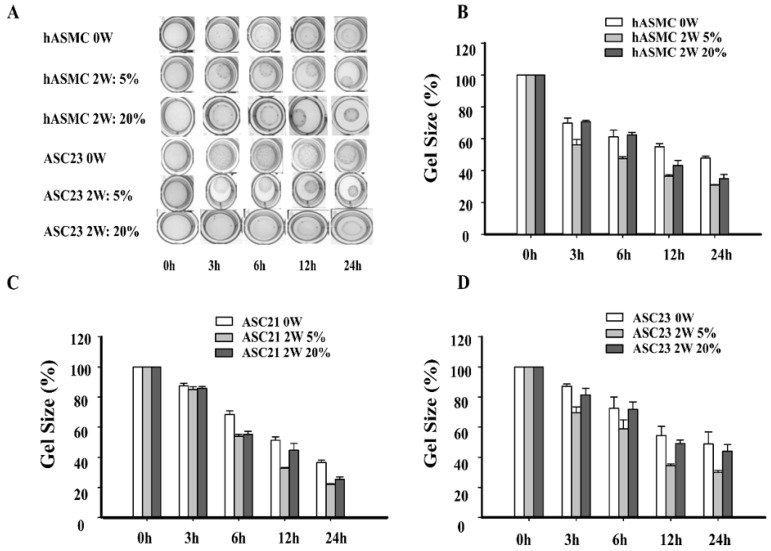
The effect of hypoxia on contractility of SMCs and ASCs. (**A**) Collagen gel lattices containing undifferentiated or differentiated SMCs and ASCs were cultured for 24 h and photographed at the indicated time points after induction with carbachol. (**B**) The relative area of gel matrices in SMCs. (**C**) The relative area of gel matrices in ASC21. (**D**) The relative area of gel matrices in ASC23. Data was expressed as means ± SE. Statistical analysis showed differences between cell cultures, oxygen levels, and time of differentiation to be significant (*p <* 0.01 for all).

Undifferentiated SMCs and ASCs showed basic contraction in a time dependent manner, reaching a minimum gel size after 24 h (SMC = 48.0%; ASC21 = 35.0; ASC23 = 50.0%). By contrast, cells differentiated in 20% oxygen conditions showed increased gel contraction after 24 h (SMC = 34.8%; ASC21 = 26.9%; ASC23 = 44.5%) (Figure 5A). Cells differentiated in 5% oxygen conditions showed the largest contraction effect (SMC = 29.4%; ASC21 = 22.3%; ASC23 = 28.9%) (Figure 5). The smooth muscle control cells differentiated in 5% oxygen showed the largest initial contraction after 3 h with a mean contraction to 53.3% of the initial gel size. Similarly, the two adipose stem cell cultures showed the largest gel contraction for the 5% oxygen cells, albeit at a slower pace than the control cells, Figure 5B–D.

Statistical analysis showed O_2_ levels (*p <* 0.01), time of differentiation (*p <* 0.01), and all cell cultures (*p <* 0.01) to be significantly different.

## 3. Discussion

There has been no previous data on the effect of hypoxia on the differentiation of SMCs from ASCs. Our study is the first study to show that a 5% oxygen concentration enhanced the differentiation of ASCs to form contractile SMCs and confirmed that functional SMCs could be produced from ASCs in vitro with a combination of TGF-β1 and BMP4. Expression of SMC-specific genes and proteins, single cell contraction assay, and collagen gel lattice assay all indicate 5% oxygen as the optimal oxygen environment during differentiation.

In general, environmental factors play a critical role in differentiation of mesenchymal stem cells, and oxygen is an important factor in non-genetic influencing factors. Our findings are compatible with previous studies that showed a similar pro-differentiation effect of hypoxia in chondrogenesis [15] and pro-angiogenic potential of human ASCs [14]. This indicates that 5% oxygen in general is able to promote differentiation processes involving ASCs. However, this deviates with previous studies on the effect of hypoxia on differentiation to non-smooth muscle cells, as a previous study showed 2% oxygen strongly inhibited chondrogenesis and osteogenesis whilst another study found hypoxia (5%) promoted chondrogenic differentiation of human ASCs, but inhibited osteogenic differentiation [12,16]. These results may not be conflicting but highlight the differential regulatory role of levels of oxygen on the differentiation processes of ASCs. Our previous results demonstrate that 15% oxygen provides the most suitable environment for inducing chondrogenesis in ASCs and it was reported that oxygen tension provides metabolic programming to direct the chondrogenic differentiation program into either permanent articular-like cartilage or hypertrophic cartilage [17]. It is possible that a similar programming will be required for smooth muscle cell differentiation whereby oxygen level needs to be altered depending on stage of differentiation and suitable phenotype. It should also be noted that a 5% oxygen level is the threshold of HIF-1A (Hypoxia-inducible factor 1-alpha) activation and lower oxygen was shown to inhibit differentiation of trophoblast giant cells, which was relieved in a stepwise fashion with increasing oxygen levels [18]. Since we also see a lower differentiation effect at 2% oxygen in combination with BMP4 and TGF-β1, there must be a more complex interplay between the TGF-Smad pathway and the HIF-1A pathway than just mere activation. 

It has been shown that heparin can induce the differentiation of ASCs into SMCs [6]. However, this requires 6 weeks of induction. Compared to this method, our data indicated that using the combination of TGF-β1 and BMP4 shortens the effective induction time to two weeks. Furthermore, the gene expression of specific contractile proteins (α-SMA, calponin, caldesmon, and MHC) was increased after 2 weeks induction of cells. Differentiation of ASCs by TGF-β1 increases expression of smooth muscle alpha actin (α-SMA) [19] whilst bone morphogenetic protein (BMP) signaling pathway induces contractile phenotype and SMC-specific genes transcription and prevents dedifferentiation of arterial SMCs [8]. The combination of TGF-β1 and BMP4 is likely to promote cell changes towards smooth muscle cells [7] and this study showed hypoxia further stimulates the differentiation process. Our group has shown previously that prolonged growth of adipose stem cells in 5% oxygen enhances vascular endothelial growth factor (VEGF) expression and decreases apoptosis [14]. Vascular endothelial growth factor was also shown to increase the expression of α-SMA and initiate cell contraction measured in a gel contraction assay [20]. It is possible that hypoxia enhances the effect of TGF-β by an autocrine stimulation by VEGF acting on the VEGF receptors. However, the precise molecular pathways are complex, as are the tissue locations and origins of smooth muscle. There have been many suggested pathways that underpin the differentiation of contractile smooth muscle cell in studies exploring regenerative techniques as well as investigating pathological process of diseases, and further studies are needed to identify the ideal combination of cytokines, optimize their dose, and determine how other elements of conditioning affect the differentiation [7,21].

## 4. Materials and Methods

### 4.1. Isolation and Culture of Human Adipose Tissue-Derived Stem Cells

Adipose tissue was obtained from two healthy patients (ASC23 from 42-year-old-female and ASC21 from 52-year-old male) undergoing liposuction procedure at the Grymer Privat Hospital (Arhus, Denmark). The clinical protocol was approved by the regional Committee on Biomedical Research Ethics of Northern Jutland, Denmark (project No. VN 2005/54). Adipose stem cells were isolated as previously described with a slight modification [22]. Briefly, adipose tissue was washed intensively with pre-warmed Dulbecco’s phosphate-buffered saline (PBS) and then digested with 65 U/mL crude collagenase (Wako, Richmond, VA, USA) at 37 °C for 60 min. The digested lipoaspirate was then centrifuged at 400× *g* for 10 min to obtain the stromal vascular fraction (SVF) pellet, which was further filtered through a 70-µm mesh cell strainer (BD Bioscience, Broendby, Denmark) after lysis of erythrocytes. The SVF was incubated overnight in a standard incubator. Culture medium was Dulbecco’s modified Eagle culture medium supplemented with 10% fetal bovine serum (FBS) 100 IU/mL penicillin, 0.1 mg/mL streptomycin, and 0.05 mg/mL gentamicin (Invitrogen, Leiden, The Netherlands). After 24 h, the non-adherent mononuclear cells were removed. The medium was changed twice a week. When reaching 80% confluence, cells were passaged or cryopreserved for further experiments, and ASCs of passage 6–10 were used in the subsequent studies. These cell cultures (ASC23 and ASC 21) have previously been extensively characterized in our laboratory, both under normoxic and hypoxic conditions [23,24,25,26].

Human aortic smooth muscle cells (SMCs) were used as control. All culture medium reagents and SMCs were purchased from Cascade Biologics (Portland, OR, USA) unless otherwise stated. Human aortic smooth muscle cells were cultured in medium 231 supplemented with smooth muscle growth supplement according to the manufacturer’s protocol. Medium was changed twice a week. The cells were passaged or cryopreserved for further experiments when reaching 80% confluence. All experiments were performed with cells of passages 6 to 10 in this study.

### 4.2. The Effect of Hypoxia on the Differentiation of SMCs and ASCs

Human aortic smooth muscle cells were cultured in medium 231 supplemented with smooth muscle growth supplement at a density of 2.5 × 10^3^ cells/cm^2^. After reaching sub-confluence, SMCs were further differentiated in medium 231 supplemented with smooth muscle differentiation supplement for 2 weeks at 2%, 5%, 10%, and 20% oxygen concentrations, respectively. Adipose-derived stem cells were seeded at a density of 5 × 10^3^ cells/cm^2^ in low glucose Dulbecco’s modified Eagle medium supplemented with 10% FBS, 100 IU/mL penicillin, 0.1 mg/mL streptomycin, and 0.05 mg/mL gentamicin until sub-confluence. Next, ASCs were further induced with 5 ng/mL TGF-β1 and 2.5 ng/mL BMP4 (R&D Systems, Abingdon, UK) for 2 weeks at different oxygen levels. The combination of TGF-β1 and BMP4 was used based on a previous report [7]. The culture medium was changed twice a week. All hypoxic culture experiments were conducted at 37 °C in a hypoxic workstation (Xvivo; BioSpherix, Redfield, NY, USA) in an atmosphere containing 5% CO_2_ balanced with nitrogen to reach oxygen concentrations of 2%, 5%, and 10%. Normoxic cell culture was performed in a standard incubator at 37 °C in a humidified atmosphere containing 5% CO_2_ and 20% oxygen. Cell characterization and functional evaluation were performed after 2 weeks.

### 4.3. Real Time Reverse Transcription Polymerase Chain Reaction (RT-qPCR)

Total cellular RNA was extracted from cells at indicated time points using an Aurum total RNA mini kit (Bio-Rad, Copenhagen, Denmark) according to the manufacturer’s instructions. The purity and concentration were determined on the basis of the 260/280 optical density (OD) ratio using spectrophotometry (NanoDrop; Thermo Fisher Scientific, Wilmington, DE, USA). Complementary DNA (cDNA) was synthesized using iScript cDNA synthesis kit (Bio-Rad, Copenhagen, Denmark). All primer sequences were designed using software Primer3 and were produced by DNA Technology (Aarhus, Denmark). PCR reactions were monitored and performed on a My-Cycler real-time PCR system (Bio-Rad, Copenhagen, Denmark). IQ SYBR Green Supermix (Bio-Rad, Copenhagen, Denmark) was used in each reaction. Reactions were performed for 40 amplification cycles as follows: 3 min at 95 °C, 10 s at 95 °C for DNA denaturation and 30 s for annealing and extension at respective annealing temperature (Table 1). The expression level for each gene of interest was normalized to that of the β-actin. The relative ratio of each gene was obtained by dividing the mRNA expression at week 2 by that of week 0. A blank without cDNA was included as a negative control.

### 4.4. Immunofluorescence Staining

Cells seeded at density of 2.5 × 10^3^ per cm^2^ in Lab-Tek II 8-well chamber slides (Nalge Nunc, Rochester, NY, USA) were fixed in 4% formaldehyde for 10 min at room temperature. After washing cells in PBS three times, cells were subsequently incubated with ice-cold methanol for 20 min at −20 °C. The following primary antibodies diluted 1:200 including mouse monoclonal anti-α-SMA and mouse monoclonal anti-MHC (Santa Cruz Biotechnology, Aarhus, Denmark) were applied. After reaction with primary antibodies at 4 °C overnight, the cells were washed with PBS three times. Alexa 488-conjugated goat anti-mouse secondary antibody (Life Technologies Europe, Naerum, Denmark) was used to detect the localization of different antibodies respectively, and cell nuclei were counterstained with Hoechst 33342. The images were acquired and processed using the microscope Axio Observer Z1 (Carl Zeiss, Göttingen, Germany). 

### 4.5. Quantitative Carbachol Contraction Assay

Single cell contraction assays were performed as previously described with a slight modification [27]. Briefly, SMCs and ASCs were seeded and grown as described in Section 4.2, in 24 well culture plates, washed with PBS, and partially detached from the bottom of culture dish after adding a non-enzymatic cell dissociation buffer (Sigma-Aldrich, Broendby, Denmark) in each well at 37 °C for 30 min. The non-enzymatic cell dissociation buffer was replaced with PBS for 30 min in order to keep the cells stable. The cell contraction was induced with 100 µM carbachol, and after 0, 5, 10, and 20 min, the images were recorded with a microscope Axio Observer Z1. The surface areas of cells at different time points were quantified using NIH Image J software (NIH, Bethesda, MD, USA). The relative area of a cell was defined as the surface area at the indicated time point divided by the surface area at time 0.

### 4.6. Effect of Hypoxia on Contractile Function

Collagen gel lattice contraction assays were performed as described previously with a slight modification [28]. Briefly, a collagen gel solution (3 mg/mL) was obtained by dissolving 100 mg rat-tail collagen type I powder (Sigma-Aldrich, Broendby, Denmark) in 16.7 mL of 0.2% acetic acid and then diluted with the same amount of sterilized water. The cells were trypsinized with 0.25% trypsin-EDTA and resuspended in culture medium at a density of 1.5 × 10^5^ cells/mL. A mixture of 400 µL well-mixed cell suspension and 200 µL of 3 mg/mL collagen solution was neutralized to pH 7.0 with 3 µL of 1 M NaOH. Then the 500 µL cell/collagen mixture was transferred to 24 well culture plates for 20 min to polymerize the collagen cell lattices. The lattices were released by gently pipetting medium at the lattice-dish interface and collagen gel contraction was initiated. Immediately 500 µL serum-free medium including carbachol (1 mM) was added to stimulate cell contraction, and gels were incubated for an additional 24 h. Images of the gel lattices were taken at 0, 3, 6, 12, and 24 h. For the groups subjected to differentiation under hypoxia, the whole experiment was performed in a hypoxic workstation. The area of gel lattices was analyzed using NIH Image J software. Quantitative results were interpreted as the percentage of the area of the gel lattice at indicated time points divided by the initial area of the lattice at 0 h. 

### 4.7. Statistical Analysis

The data were analyzed using SPSS 22.0 software (IBM, Armonk, NY, USA). All quantitative data were analyzed by univariate analysis of variance. Tukey’s test of significance was used in the post-hoc analysis. Data are presented as mean ± standard error (SE). All experiments were performed with two ASC samples and one SMC sample and repeated at least three times. 

### 4.8. Ethics

Retrieval of human specimens and all protocols were approved by the regional committee on biomedical research ethics of Northern Jutland, Denmark (project No. VN, date 5/4/2005). 

## 5. Conclusions

Hypoxia influences differentiation of smooth muscle cells from adipose stem cells and 5% oxygen was the optimal condition to generate smooth muscle cells that contract from adipose stem cells.

## Figures and Tables

**Table 1:** Genes and primer sequences used in real time qPCR analysis.

Gene	Forward Primer Sequence	Reverse Primer Sequence	Annealing Temperature (°C)
β-actin	5′-ATC ATG TTT GAG ACC TTC AA-3′	5′-AAA GCC CTG GAA CTT GAC C-3′	58
α-SMA	5′-AGC AGC CCA GCC AAG CAC TG-3′	5′-AGC CGG CCT TAC AGA GCC CA-3′	60
Calponin	5′-CTG GCT GCA GCT TAT TGA TG-3′	5′-CTG AGA GAG TGG ATC GAG GG-3′	60
Caldesmon	5′-TCT GAG CCT TCT GGT TGG TC-3′	5′-CCT CGG GAA GAA GTT TCA GA-3′	60
SM-MHC	5′-AAA GCC CTG GAA CTT GAC C-3′	5′-AGA TTT TGC TCT GCC CTA TCC-3′	60

## Abbreviations

SMC	Smooth muscle cell
ASC	Adipose tissue-derived stem cell
α-SMA	Alpha-smooth muscle actin
MHC	Myosin heavy chain
PBS	Phosphate-buffered saline
SVF	Stromal vascular fraction
FBS	Fetal bovine serum
SMC	Smooth muscle cell
TGF-β1	Transforming growth factor beta 1
BMP	Bone morphogenetic protein
BMP4	Bone morphogenetic protein 4
VEGF	Vascular endothelial growth factor
RT-PCR	Real time reverse transcription polymerase chain reaction
OD	Optical density
cDNA	Complementary DNA
SEM	Standard error of the mean
